# Liposomal bupivacaine for the management of postsurgical donor site pain in patients with burn injuries: a case series from two institutions

**DOI:** 10.1002/ccr3.1292

**Published:** 2017-12-05

**Authors:** Sharmila Dissanaike, Jayne McCauley, Carlo Alphonso

**Affiliations:** ^1^ Department of Surgery Texas Tech University Health Sciences Center Lubbock Texas; ^2^ US Army Institute of Surgical Research Fort Sam Houston Texas

**Keywords:** Acute pain, analgesia, burns, pain, postoperative, skin transplantation

## Abstract

Donor site pain associated with skin graft procedures is frequently intense and difficult to treat. Liposomal bupivacaine, a prolonged‐release local anesthetic indicated for single‐dose administration to produce postsurgical analgesia, may be a viable option in managing donor site pain.

## Introduction

Postsurgical pain management following skin graft procedures in patients with burn injuries is often difficult and requires multimodal therapy for proper wound healing and recovery 1, 2. However, skin grafting, dressing changes, and other procedures involved in treating burn wounds may produce pain that can be more intense than the pain associated with the initial burn injury itself, particularly at the graft donor site 1, 3, and frequent burn care procedures create additional distress 4, 5. Pain management in these patients is also complicated by profound physiologic changes associated with the burn injuries that can alter the patient's response to analgesic agents 2, 6. If analgesics and sedatives fail to control the pain, anxiety can worsen it 1.

Opioids are a mainstay in the treatment of wound and procedural pain in patients with burn injuries because of their analgesic efficacy 7, 8, but opioid use is also associated with nausea, vomiting, constipation, urinary retention, and more serious, potentially fatal adverse events such as respiratory depression 9, 10, 11. In addition, prolonged use of opioids may produce tolerance and hyperalgesia 12. These complications may lead to additional treatment challenges and longer hospital stays 5.

Although traditional local anesthetic formulations can provide effective and safe analgesia at the surgical site when administered as recommended, their duration of action is short, often lasting only 12 h or less 13. The use of prolonged‐release formulations such as liposomal bupivacaine (Exparel^®^; bupivacaine liposome injectable suspension, Pacira Pharmaceuticals, Inc., Parsippany, NJ) can extend the duration of postsurgical analgesia while reducing opioid requirements. Liposomal bupivacaine, a multivesicular formulation of bupivacaine, is indicated for single‐dose administration into the surgical site to produce postsurgical analgesia in adults 14. It has been shown to provide postsurgical analgesia for up to 72 h and to reduce postsurgical opioid requirements compared with bupivacaine HCl or placebo in numerous surgical settings 15, 16. However, the use of liposomal bupivacaine in the management of donor site pain has not been previously described. Analgesic efficacy following one‐time infiltration of liposomal bupivacaine into the donor sites of burn‐injured patients undergoing skin graft procedures 17, 18 was evaluated.

## Materials and Methods

### Patients

All patients underwent skin graft procedures at the Timothy J. Harnar Burn Center at University Medical Center affiliated with the Texas Tech University Health Sciences Center in Lubbock, Texas (Cohort 1) 17, and the US Army Institute of Surgical Research (USAISR) in Fort Sam Houston, Texas (Cohort 2) 18, 19. Informed consent for anesthesia and surgery was obtained, in accordance with surgical protocols at the two institutions. Informed consent for administration of liposomal bupivacaine was not required because of the retrospective nature of the research. Study descriptions and procedures were reviewed by the Clinical Research Institute and Institutional Review Board of Texas Tech University Health Sciences Center and by the Research Regulatory Compliance Division at the USAISR.

#### Cohort 1

**Figure 1:**
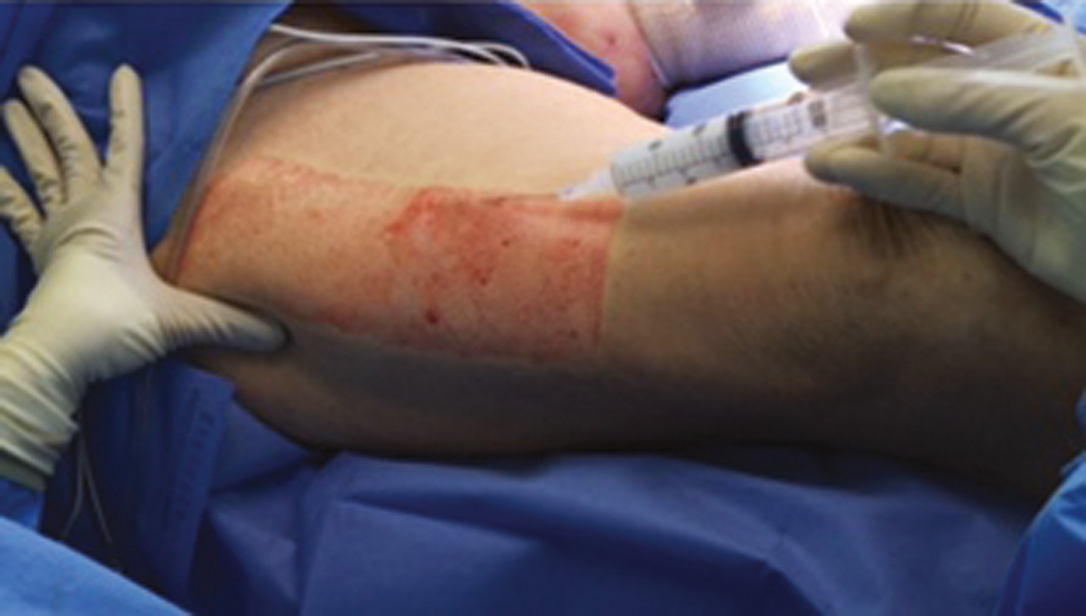
Administration of liposomal bupivacaine into a thigh donor site prior to harvesting for split‐thickness skin grafting. Photograph courtesy of Sharmila Dissanaike, MD, FACS, and Jayne McCauley, MD.

Patients in Cohort 1 underwent split‐thickness skin grafting at the Harnar Burn Center from March 2015 to August 2015. Patients whose donor site(s) were ≤10% of the total body surface area (TBSA) were included in the study. In accordance with the product label, liposomal bupivacaine 266 mg/20 mL was diluted with saline 0.9% to total volumes ranging from 45 to 120 mL to provide sufficient coverage of the surgical area 14, 20. The dose administered ranged from 89 to 266 mg. The donor sites were the thigh in 19 patients, as exemplified in Figure 1, and the arm and chest in one patient, and ranged in size from 72 to 1386 cm^2^. Standard silver‐based dressing was applied to the donor site following harvesting 17.

Amounts of intravenous and oral opioids consumed were converted to oral morphine equivalents 21. Analgesic agents used included morphine, oxycodone, hydrocodone, hydromorphone, tramadol, codeine/acetaminophen, hydrocodone bitartrate/acetaminophen, fenoprofen calcium, and ketorolac.

#### Cohort 2

Patients in Cohort 2 underwent skin graft surgery at USAISR between February 2015 and March 2015. Donor sites were 20 cm^2^–400 cm^2^. Each patient was prepared for surgery according to standard protocol. Liposomal bupivacaine 266 mg/20 mL was diluted to a total volume of up to 250 mL with normal saline to ensure adequate coverage of the donor site, admixed with Pitkin's solution (lactated Ringer's solution with 1:1,000,000 epinephrine), and infiltrated into the skin donor site prior to harvesting. Skin grafts were collected from the anterolateral thigh, and standard Xeroform™ dressings (Medtronic Minimally Invasive Therapies [formerly Covidien], Minneapolis, MN) were applied to donor sites 18, 19.

### Assessments

#### Cohort 1

Baseline information on patient and injury characteristics, pain scores, and dosages of pain medications were recorded. Pain intensity was typically recorded every 4 h and was scored using a validated 11‐point numeric rating scale (NRS) in which the patient selected an integer from 0 (“no pain”) to 10 (“worst imaginable pain”) 17, 22. Liposomal bupivacaine was administered via injection at the donor site while the patient was sedated under general anesthesia in the operating room, thereby blinding the patients and their bedside nurses to the intervention to avoid bias during pain assessments. Opioid consumption was recorded in morphine equivalents on postoperative days (PODs) 1, 2, and 3. Postsurgical medication use was compared with the amount consumed the day before surgery with a Student's *t*‐test using the T.TEST function in the Microsoft Excel software package (Microsoft, Redmond, WA) 17.

#### Cohort 2

Demographic data and injury characteristics were recorded for each patient, and patients reported postsurgical pain at the donor site using the Defense and Veterans Pain Rating Scale (DVPRS) at 1, 4, 8, 12, 24, 36, 48, and 72 h after surgery 19. The DVPRS is a validated instrument that uses an 11‐point NRS to quantify pain from 0 (“no pain”) to 10 (“as bad as it could be, nothing else matters”), functional word descriptors, visual cues in the form of green‐yellow‐red traffic color codes, and facial expressions 23, 24. Donor sites were assessed for complications, and patients were monitored for other adverse events 18.

## Results

### Cohort 1

Cohort 1 included 20 patients ranging in age from 18 to 68 years (mean age: 41 years); 13 patients (65%) in the cohort were male. The dose of liposomal bupivacaine administered to each patient ranged between 89 and 266 mg (mean dose: 231 mg). The mean size of the burn area was 12% TBSA (range: 1–50% TBSA). Because the mean area of the patients’ donor sites was 441 cm^2^, the average dose of liposomal bupivacaine administered was 0.52 mg/cm^2^.

The mean patient‐reported pain scores declined steadily over POD 1 through POD 3 but were not significantly different from the pain scores recorded the day before surgery, as shown in Table 1 17. Similarly, the mean amounts of morphine equivalents consumed also decreased during the first 3 days following surgery and were not significantly different from the mean amount of morphine equivalents consumed the day before surgery. One‐quarter of the patients (5 of 20) reported little to no pain at the donor site by POD 3 (range, NRS scores: 0 through 2), and many, when asked, were unaware that they had had surgery at that site until the dressings were removed. None of the patients experienced complications at the donor site 17.

**Table 1:** Cohort 1: Pain scores and consumption of pain medication. Reprinted with permission from McCauley et al. 17

	Day before surgery	POD 1	POD 2	POD 3
Pain score^a^				
Mean (range)	4.5 (0–8.3)	5.0 (3.0–8.2)	4.3 (0–7.5)	3.7 (0–7.0)
*P* Value^b^		0.39	0.82	0.25
Morphine equivalents, mg				
Mean (range)	34.4 (4.0–66.3)	33.0 (3.0–94.4)	29.6 (0–94.4)	29.3 (2.5–94.4)
*P* Value^b^		0.84	0.53	0.5

POD, postoperative day.

a Pain was rated on a validated 11‐point numeric rating scale where 0 = “no pain” and 10 = “worst imaginable pain imaginable”.

b Comparison of each POD versus the day before surgery.

### Cohort 2

Cohort 2 consisted of five patients, four males, and one female, with a mean age of 32 years (range: 20–54 years). The mean size of the donor site area was 184 cm^2^, as shown in Table 2 18, 19. The four male patients had a mean burn area of 7% TBSA, which ranged from 1% to 22%; the extent of the burn area for the female patient was not available 19.

**Table 2:** Cohort 2: Pain Scores. Reprinted with permission from Alphonso et al. 18; Alphonso and Leazer 19

Patient	Donor site area (cm^2^)	DVPRS score after surgery^a^							
		1 h	4 h	8 h	12 h	24 h	36 h	48 h	72 h
1	100	0	0	0	0	0	0	0	0
2	20	0	0	3	0	Discharge^b^	–b	–b	–b
3	200	0	0	0	–c	0	0	0	–c
4	400	0	5	Asleep^c^	0	0	5	0	0
5	200	0	0	Asleep^c^	0	0	0	0	Discharge^b^

DVPRS, Defense and Veterans Pain Rating Scale; h, hour.

a Pain was rated on the DVPRS, a validated 11‐point numeric rating scale where 0 = “no pain” and 10 = “”as bad as it could be, nothing else matters”.

b Donor site pain score was not obtained because the patient had been discharged.

c Donor site pain score was not obtained for unknown reason(s) or the patient was sleeping.

During the first 72 h after surgery, three patients reported no postsurgical pain (i.e., DVPRS score = 0) at time points when a pain score was recorded. In the other two patients, the pain score was 0 at most time points. DVPRS scores >0 were observed at 8 h after surgery in one patient and at 4 and 36 h after surgery in another patient, but those scores returned to 0 during subsequent time points (Table 2) 18, 19. No complications were reported for any of the patients’ skin graft surgeries, and all donor sites healed properly with no signs of inflammation, hemorrhage, or infection. Finally, the intervention was well tolerated by the patients, and no adverse effects were reported.

## Discussion

Infiltration of liposomal bupivacaine into the graft donor sites in burn‐injured patients undergoing skin grafting was well tolerated and provided effective postsurgical analgesia at the donor site for up to 72 h, as reflected in the pain scores at the donor site observed during the first 3 days after surgery. The amount of postsurgical opioid pain medication consumed by patients in Cohort 1 was not significantly different from presurgical levels 17, further supporting the efficacy of liposomal bupivacaine in this setting.

Effective pain management is crucial for optimal wound healing and the psychological well‐being of patients 1, and donor site pain may be far more intense than pain at the site of the graft in many patients. There is currently no standard procedure for managing pain at the donor site 3; liposomal bupivacaine may offer a promising new postsurgical analgesic option for burn‐injured patients.

Opioid analgesics are frequently used in the management of procedural pain in patients with burn injuries 5, 25, despite the widely recognized burden of opioid‐related adverse events, overuse, and misuse. Nonsteroidal anti‐inflammatory drugs, benzodiazepines, antidepressants, gabapentin, ketamine, and *α*_2_‐adrenoceptor agonists are potentially useful adjuvants in the treatment of pain in these patients 1, 2, 26.

The role of local anesthetics as alternatives to opioids in the management of pain in burn‐injured patients is not as well established, but several studies 3, 6, 26, 27, 28, 29 support their efficacy and safety for pain management in those patients. Continuous local anesthetic infusion 3, 28, 29 and single‐injection nerve block 28 have been shown to reduce split‐thickness skin graft donor site pain. However, continuous local infusion is associated with a risk of infection, possibility of catheter displacement, and potential pump malfunction 30. The use of single‐injection peripheral nerve block overcomes these disadvantages, but is limited by its shorter duration of action (maximum of 8–24 h) 31. Liposomal bupivacaine offers a viable alternative to these techniques by providing effective, prolonged postsurgical analgesia after a single administration 32 without the use of indwelling catheters. Topical application of lidocaine has also been shown to be effective for controlling skin graft donor site pain 6, 26. The utility of traditional local anesthetics is limited, however, by their relatively short duration of action 13; accordingly, pain in the studies of lidocaine applied topically to donor sites was assessed for ≤12 h 6, 26. In contrast, pain assessments in burn patients given liposomal bupivacaine extended through POD 3 (Tables 1 and 2). Systemic absorption following the application of topical lidocaine to areas where skin integrity has been compromised (e.g., by harvesting) has not been extensively characterized. As high plasma concentrations of lidocaine may produce central nervous system and cardiac toxicity 13, the systemic absorption of topical lidocaine for the management of donor site pain merits further research. In contrast, human systemic plasma concentrations of liposomal bupivacaine from several surgical settings have been measured through 96 h after administration of 106, 266 (the highest US Food and Drug Administration–approved dose), 399, and 532 mg of liposomal bupivacaine and compared with plasma concentrations of bupivacaine HCl 100 mg at the same time points 33. There has been no evidence of cardiac or neurological toxicity in any of the clinical studies of liposomal bupivacaine reported to date 34. When preparing to administer liposomal bupivacaine, skin integrity and thickness were not the key considerations, but rather size of the donor surgical area and the degree of dilution needed to provide sufficient coverage of the donor area. Data from studies of liposomal bupivacaine in other surgical settings have shown that total dose, route of administration, and vascularity of the administration site affect systemic absorption 14.

In general, the analgesic efficacy of intravenous local anesthetics for managing donor site pain has been difficult to assess because of differences in study interventions, such as the loading dose, infusion rates and periods, and relevance of body weight in determining dosage 27. Intravenous lidocaine was superior to placebo in analgesic efficacy during dressing changes and/or debridement in burn‐injured patients in a randomized, double‐blind crossover study. However, the same study showed that intravenous lidocaine was not significantly different from placebo in the frequency of usage of patient‐controlled analgesia, the amount of morphine consumed, or the level of satisfaction with the pain control and the procedure during the first and second dressing changes. The current study did not include a placebo control group, and assessments of patient‐controlled analgesia usage and patient satisfaction were not performed. However, opioid consumption decreased during the first 3 days following surgery and was not significantly different from the day before surgery (Table 1).

### Study limitations

The primary limitations of this preliminary study were the small sample size of the patient cohorts and selection bias (i.e., donor site ≤10% of the TBSA as an inclusion criterion for Cohort 1), which make it difficult to generalize these findings to a larger population of burn‐injured patients with a greater variation in baseline comorbidities, opioid history, and burn size and severity. Variability in the dilution and dose of liposomal bupivacaine administered may have contributed to interpatient differences in analgesic efficacy. Clinicians have the flexibility to admix liposomal bupivacaine with bupivacaine HCl (using a dose of bupivacaine HCl that is below 50% of the liposomal bupivacaine dose) prior to administration 35. The admixture is well tolerated and may potentially shorten the time to onset of analgesia; however, clinical studies are needed to assess the efficacy and safety of admixing the two formulations 35. Additional limitations included the heterogeneity in the type and doses of opioid medications used by different patients, limitation of pain assessments to ≤72 h after surgery, and the inherent limitations of subjective measures such as pain scores.

Although the data presented here are preliminary in nature, they support further investigation into the use of liposomal bupivacaine in burn patients undergoing skin graft surgery. Because of the limited duration of pain assessments, it would be desirable to include later time points in future studies to determine whether this agent can control pain beyond 3 days. Future research that is controlled for concomitant pain medication, comorbid conditions, and risk factors could help better define patients that may benefit most from this treatment.

## Conclusions

Liposomal bupivacaine may be a viable therapeutic option for providing donor site postsurgical analgesia for burn‐injured patients undergoing skin graft procedures. This treatment was able, in some cases, to completely relieve pain during the first 3 days following surgery. The absence of adverse events or other complications indicates that liposomal bupivacaine may be well tolerated in this surgical population. Larger randomized, controlled, studies are required to further investigate the use of liposomal bupivacaine in this setting.

## Authorship

SD: involved in study concept and design; involved in the critical revision and review of the manuscript text and figures, as well as approval of the final draft for submission. JM: collected data; involved in the critical revision and review of the manuscript text and figures, as well as approval of the final draft for submission. CA: involved in study concept and collected data; involved in the critical revision and review of the manuscript text and figures, as well as approval of the final draft for submission.

## Conflict of interest

Sharmila Dissanaike, Jayne McCauley, and Carlo Alphonso have no financial or other conflicts of interest to report.
